# Editorial: Cycling: performance enhancement across the disciplines

**DOI:** 10.3389/fspor.2023.1285884

**Published:** 2023-09-29

**Authors:** Everton Crivoi Carmo

**Affiliations:** Department of Physical Education, Senac University Center, São Paulo, Brazil

**Keywords:** cycling, performance, Technology, Sport, training

**Editorial on the Topic Research** Cycling: performance enhancement across the disciplines

Dear readers,

It is with great honour that we present this Research Topic of Frontiers in Sports and Active Living, themed “Cycling: Performance Enhancement across the Disciplines,” dedicated to the fascinating and intricate world of cycling. Over the years, we have witnessed a phenomenal growth in cycling and the scientific knowledge produced across its various disciplines. The sport's growth is marked by the inaugural unified UCI World Championships, where, for the first time in history, the elite of cycling came together to compete for world titles in different disciplines.

On the other hand, it is with great sorrow that we pay tribute to Professor Louis Passfield, one of the greatest scientists in sports, who served as one of the guest editors for this Research Topic. Louis Passfield made an exceptional contribution to sports science applied to cycling. His invaluable contributions to the science of cycling have shaped our field of study and continue to inspire the next generation of scientists. There is a void in the scientific community, but his legacy will endure through the work he led and the minds he influenced.

## Cycling: a rising revolution

From the early days of competitive cycling to today's world-class competitions, we have witnessed an unrelenting pursuit of peak performance on two wheels. Cycling has expanded its boundaries beyond just roads or tracks, encompassing diverse disciplines such as cyclocross, mountain biking, BMX, and many others. Each discipline challenges human limits and demands specific scientific understanding to achieve success. The desire to surpass limits and achieve ever greater results has driven technological and scientific innovation. There is a growing demand for scientific research dedicated to the inherent complexities of different cycling characteristics and physiological demands, exploring their various facets. In this Research Topic, we delve into the complex world of cycling and performance improvement across different disciplines. The Research Topic features five original articles from different perspectives on performance.

The study titled “The relationship between pedal force application technique and the ability to perform supramaximal pedaling cadences,” conducted by Yuta Yamaguchi, Mitsuo Otsuka, Kohei Watanabe, Naoki Wada, and Tetsunari Nishiyama, provides valuable insights into the relationship between pedal force application technique and the ability to perform supramaximal pedalling cadences. The results are particularly relevant as they may indicate that maximum cadence can be a useful metric for assessing pedal force application techniques, especially for novice cyclists, without the need for expensive devices.

Continuing our exploration into the science of cycling, the study “Longitudinal alterations of pulmonary V.O2 on-kinetics during moderate-intensity exercise in competitive youth cyclists are related to alterations in the balance between microvascular O2 distribution and muscular O2 utilization” presents a notable advancement in understanding the dynamics of oxygen consumption during moderate-intensity cycling in competitive young cyclists. Led by Matthias Hovorka, Bernhard Prinz, Dieter Simon, Manfred Zöger, and Clemens Rumpl, the study investigated the dynamic adjustment of pulmonary oxygen consumption during moderate-intensity cycling over 15 months in young cyclists. Improvements in muscular oxygen supply and utilization capacity may have contributed to enhancing the dynamic adjustment of the oxidative “machine” in competitive young cyclists. Furthermore, this indicates a strong link between poor oxygen distribution in tissues and the V.O2 time constant.

The study conducted by Magnus K. Hyttel, Mathias Kristiansen, and Ernst, titled “Maximal accelerations for twelve weeks elicit improvement in a single out of a collection of cycling performance indicators in trained cyclists,” sheds light on how maximal accelerations can affect the performance of trained cyclists. The results of this study suggest that supplementary maximal acceleration training led to modest yet favourable changes in performance indicators compared to comparable control cyclists. This highlights the importance of considering different training approaches to improve cycling performance.

Assaf Yogev, Jem Arnold, Hannah Nelson, David C. Clarke, Jordan A. Guenette, Ben C. Sporer, and Michael S. Koehle conducted the article “The effect of severe intensity bouts on muscle oxygen saturation responses in trained cyclists.” The aim was to describe the effect of self-paced severe intensity bouts on the vastus lateralis (VL) muscle non-invasively measured by a wearable NIRS (Near-infrared spectroscopy) sensor and examine its reliability. This study highlights the heterogeneity in muscle oxygen saturation responses during severe intensity cycling bouts and the reliability of a wearable NIRS sensor in measuring these responses in trained cyclists. This has important implications for understanding the physiological demands of high-intensity cycling and can assist cycling professionals in optimizing training strategies.

We conclude with the article “Comparing the reliability of muscle oxygen saturation with common performance and physiological markers across cycling exercise intensity,” which sheds light on the reliability of muscle oxygen saturation (SmO2) measurements compared to other common physiological markers during cycling at different intensities. The study conducted by Assaf Yogev, Jem Arnold, Hannah Nelson, David C. Clarke, Jordan A. Guenette, Ben C. Sporer, and Michael S. Koehle highlights that SmO2, measured by a wearable NIRS device, demonstrates good to excellent reliability during a multi-stage incremental cycling test at various intensities. Furthermore, SmO2 showed similar reliability to V˙O2 and HR, being more reliable than [BLa] and RPE across the exercise intensity spectrum. This suggests that SmO2 is an appropriate tool for monitoring internal load during cycling, complementing other physiological markers.

As we continue to deepen our understanding of cycling physiology and its application in sports training, studies like these provide valuable insights for athletes and coaches in the pursuit of performance optimization. Cycling is constantly evolving, and scientific research plays a vital role in driving this evolution.

With profound gratitude for the ongoing commitment of the scientific community to cycling research, we leave you with this Research Topic.

